# Liquid Level Sensor with Two FBGs Embedded in a PDMS Diaphragm: Analysis of the Linearity and Sensitivity

**DOI:** 10.3390/s22031268

**Published:** 2022-02-07

**Authors:** Eliton Morais, Maria José Pontes, Carlos Marques, Arnaldo Leal-Junior

**Affiliations:** 1 Graduate Program in Electrical Engineering, Federal University of Espirito Santo (UFES), Vitoria 29075-910, Brazil; eliton.morais@edu.ufes.br (E.M.); mjpontes@ele.ufes.br (M.J.P.); 2 Physics Department & I3N, University of Aveiro, 3810-193 Aveiro, Portugal; carlos.marques@ua.pt

**Keywords:** fiber Bragg gratings, liquid level, silicone rubber, polydimethylsiloxane, pressure sensors

## Abstract

This paper presents a fiber optic, liquid level sensor system based on a pair of fiber Bragg gratings (FBGs), embedded in a circular silicone (PDMS—polydimethylsiloxane) rubber diaphragm. The measurement principles of this sensor, whose diaphragm structure is about 2.2 mm thick with 45 mm in diameter, are introduced. To analyze the linearity and sensitivity of the sensor, the diaphragm was subjected to compression tests as well as to liquid level loading and unloading. The force and liquid level increase tests showed that inserting two FBGs (0.99453 for force and 0.99163 for liquid level) in the diaphragm resulted in a system with greater linearity than that with individual FBGs. This occurred where FBG1 showed 0.97684 for force and 0.98848 for liquid level and FBG2 presented 0.89461 for force and 0.93408 for liquid level. However, the compression and water level decrease tests showed that the system (*R^2^* = 0.97142) had greater linearity with FBG2 (0.94123) and lower linearity with FBG1 (0.98271). Temperature characterization was also performed, and we found that sensitivity to FBG1 temperature variation was 11.73 pm/°C and for FGB2 it was 10.29 pm/°C. Temperature sensitivity was improved for both FBGs when compared with uncoated FBGs with typical values of 9.75 pm/°C. Therefore, the proposed FBG-based sensor system is capable of simultaneous measurement of force and temperature in a compact diaphragm-embedded system.

## 1. Introduction

Fiber optic sensing technology has a huge potential to be used in the industry [1], in health [2], in radioactive environments [3], in explosive environments [4], and in structural health monitoring [5]. In recent years, fiber optic sensors have increasingly expanded due to several benefits, including their immunity to electromagnetic interference, small size, lightweight, high sensitivity, multiplexing capacity, and their ability to transmit signal light and operate with relatively modest optical power. Such fiber optic sensors are also intrinsically safe when compared with conventional electrical sensors’ systems in explosive environments [6,7].

Fiber optic sensors can be also applied to detect liquid levels [7]. Since fiber sensors have many benefits and uses. Various fields have a growing demand for them, such as the chemical industry for explosive fuel detection [8], wastewater treatment plants [9], and flood warning [10]. Nowadays, different types of structures and fiber configurations are used to customize specific applications, such as measuring structural and industrial parameters (liquid level, pressure, temperature, humidity, and deformation, among others) [6].

Among several types of sensors that measure the optic fiber liquid level, we emphasize interferometric fiber optic sensors and fiber Bragg grating (FBG) sensors [7]. Interferometers have been widely studied because of their wide dynamic range, high accuracy, and high sensitivity. They utilize the interference between two beams propagated by different optical paths of one or two fibers [11] and are classified into four types, called Fabry–Perot, Mach–Zehnder, Michelson, and Sagnac [7]. A fiber Bragg grating is produced by a modulation of the core refraction index when the fiber is exposed to a periodic intensity light pattern [12]. FBG works as a reflective spectral filter that selects a specific wavelength, e.g., it is a mirror that reflects a certain wavelength and transmits the other wavelengths [7]. The main difference that determines the use of FBGs in this project is their ability to multiplex several sensors in just one fiber, a feature that could not be accomplished with fiber sensors based on interferometers [9].

Pressure can be measured using FBGs without any coating or structure to assist the measurement. However, to make FBGs viable to this application, since its typical pressure sensitivity is about 3.04 pm/MPa, indirect detection strategies should be considered. It qualifies the FBG sensors to meet the pressure measurement accuracy requirements in practice [13,14,15]. The sensitivity of pressure measurement can be improved by doing pressure detection indirectly through strain detection [13,15,16]. For this, FBG is incorporated in composites [17], polymers [18,19] wood [20] and metal alloys [21], diaphragm-cantilevers [13,22], Bourdon tubes [23], or to a diaphragm structure [24], among others. The structures formed enable the measurement of different parameters, such as pressure [25], vibration [26], liquid level [27], and concentration of chemical compounds [28]. However, some of the structures presented are relatively complex to manufacture, and multiple sensors are difficult to be multiplexed in a single optical fiber [14,15]. A relatively simple construction approach that is often employed for monitoring a liquid level is the use of a diaphragm with built-in FBGs. This setting estimates the liquid level from the hydrostatic pressure applied to the diaphragm. However, this method only works for a single fluid, and it must have a constant relative density [9]. In addition to liquid level monitoring, diaphragm-based sensors with built-in FBGs are employed in sensing pressure [25], vibration [26], acceleration [29], and force [30].

FBGs are sensitive to temperature and mechanical stress, since these effects shift the Bragg wavelength peak due to the thermo-optical effect with thermal expansion and effective strain-optic constant. For FBGs embedded in a diaphragm, this stress comes from the diaphragm, which undergoes tension because of the liquid level [7,31,32]. Therefore, it is important to consider the geometry and characteristics of the diaphragm material, since it not only is deformed by liquid pressure but also can suffer thermal changes from the liquid being measured [32]. Thus, different types of materials have been studied for the construction of sensors based on FBGs inserted in diaphragms, such as epoxy resin [31], nitrile rubber [32], carbon composites [24], graphene [33], and natural rubber [34]. Changes caused by temperature are undesirable for liquid level measurement. For systems with a constant temperature, this will not cause measurement errors. However for varying temperatures, another FBG sensor can be installed and isolated from pressure variations to only perform the measurement of the temperature, thus reducing cross-sensitivity [31,32]. However, the influence of temperature on the mechanical properties of the diaphragm should also be analyzed to find the cross-sensitivity compensation for high temperatures [9]. Recently, FBG pressure sensors embedded or attached to diaphragms have been studied and widely implemented. Leal–Junior et al. [9] developed a sensor created from a polyurethane diaphragm with two FBGs embedded so that they are positioned in opposite directions, considering the neutral line of the diaphragm fold as a reference. Thus, the sensor showed high sensitivity and linearity for all analyzed cases; it was also possible to perform temperature compensation. However, the multiparameter analysis was not performed. Her et al. [35] developed a sensor for measuring pressure and water level built from an FBG integrated in an epoxy diaphragm, where the effect of diaphragm thickness on sensor sensitivity and accuracy was investigated. Nevertheless, this sensor needed a temperature compensation for practical applications.

We can observe that the use of two FBGs in one diaphragm has been little studied. Therefore, this study aims to analyze the linearity and sensitivity of a liquid level sensor when subjected to compression and immersion tests in a water tank. The sensor was built from two FBGs inserted into a silicone rubber diaphragm (PDMS—polydimethylsiloxane). The FBGs were positioned in opposite directions, considering the diaphragm bending’s neutral line as a reference. The goal is to demonstrate the sensitivity for multiparameter sensing in a compact diaphragm-embedded FBG sensor system. Thus, this analysis provides guidelines on the embedment of two FBGs in the diaphragm with the discussion of the sensor behavior under different parameters. This can be used for other purposes that need a fiber optic sensor incorporated into a diaphragm, such as detection of pressure, vibration, acceleration, and force [6,36].

## 2. Operating Principle and Experimental Configuration

### 2.1. Operating Principle

FBG is often used in telecommunication and sensing technologies [37] and is made from a periodic or almost periodic modulation of the refraction index within the core of a photosensitive optical fiber [37,38]. FBG typically works as a spectral filter, so that when broadband light is propagated along the grating, the only wavelength that meets Bragg’s resonance condition is reflected [30,37]. The reflected wavelength is called a Bragg wavelength (*λ_B_*). It shows linearity with the effective refractive index (*n_eff_*) and the period (Λ), as shown in Equation (1) [39].

(1) $$\lambda_{B}=2n_{eff}Ʌ$$

Reflected wavelength is mainly affected by the effective refractive index and the grating period [13]. These are influenced by external disturbances, including temperature and stress, thus displacing the central wavelength and affecting the reliability and sensitivity of the sensor [14]. In short, if stress is applied to an FBG, it will change Bragg’s wavelength behavior due to variations in the grating period. This variation also happens when the optical fiber in which the FBG is recorded suffers thermal expansion because of temperature variations. The variation in Bragg’s wavelength is caused by changes in the refractive index and happens with the thermo-optic effect, which is caused by temperature variation. Wavelength is also affected by the photoelastic effect, caused by fiber stress [16]. The effects of distortion and temperature variation on the wavelength deviation of the FBG center are expressed by Equation (2) [13].

(2) $$\Delta \lambda_{B}=\lambda_{B}\left[\left(1-P_{\varepsilon }\right)\varepsilon +\left(\alpha +\zeta \right)\Delta T\right],$$

where *P_ε_* is the effective photoelastic constant, ε is the stress in the fiber, *α* is the coefficient of thermal expansion, *ζ* is the thermo-optical coefficient, *λ_B_* is the initial Bragg wavelength, and Δ*T* is temperature variation.

FBG response as a function of temperature and stress depends not only on the physical parameters of the optical fiber, but also on the diaphragm’s properties and its geometrical parameters. These parameters influence the diaphragm’s deflection, which then passes to the optical fiber [16], because when pressure is applied on the flat diaphragm, that flexion passes to the FBGs, thus displacing the wavelength of the fiber network [14].

However, as FBGs have a cross-sensitivity between stress and temperature, they are inclined to errors during liquid level measurement [31]. They can only be used under constant temperature, except when a compensation technique is used to isolate the effects of temperature on the measurements. Temperature sensors are commonly used as a compensation technique since they are also based on FBG to estimate the difference between the temperature sensor and the liquid level sensor [31,32].

The hydrostatic pressure caused by the variation of liquid level is what causes pressure variation in the diaphragm. Thus, we can assume that *(*1 *− P_ε_) ε* is level sensitivity (*k_L_*) and *(α + ζ)* Δ*T* is temperature sensitivity (*k_T_*). Therefore, we can rewrite Equation (2) by associating the initial Bragg wavelength (*λ_B_*_,0_), temperature variation (Δ*T*), and level variation (Δ*L*) with the Bragg wavelength variation [31].

(3) $$\lambda_{B,L}=k_{L}\Delta L+k_{T}\Delta T+\lambda_{B,0}$$

This equation can be used for both FBGs inserted into the diaphragm. For the FBG that measures temperature, the *k_L_*Δ*L* term equals zero. Equations (4)–(6) refer to the Bragg wavelength measured from FBG1 (*λ_B_*_1*,L*_), FBG2 (*λ_B_*_2*,L*_), and temperature FBG (*λ_B,T_*), respectively.

(4) $$\lambda_{B1,L}=k_{L1}\Delta L+k_{T1}\Delta T+\lambda_{B1,0}$$

(5) $$\lambda_{B2,L}=k_{L2}\Delta L+k_{T2}\Delta T+\lambda_{B2,0}$$

(6) $$\lambda_{B,T}=k_{TT}\Delta T+\lambda_{BT,0}$$

where Δ*L* is liquid level variation, Δ*T* is temperature variation, *k_L_*_1_ is the FBG1 sensitivity to level variation, *k_L_*_2_ is the FBG2 sensitivity to liquid level variation, *k_T_*_1_ is the FBG1 sensitivity to temperature variation, *k_T_*_2_ is the FBG2 sensitivity to temperature variation, *k_TT_* is the FBG temperature sensitivity to temperature variation, and *λ_B_*_1,0_, *λ_B_*_2,0_, and *λ_BT_*_,0_ are the initial wavelengths of FBG1, FBG2, and FBG temperature, respectively [32].

If we have sensitivity values, we can correlate the equations above to find the variation of liquid level and the variation of applied force in the sensor.

### 2.2. Experimental Configuration

**Figure 1:**
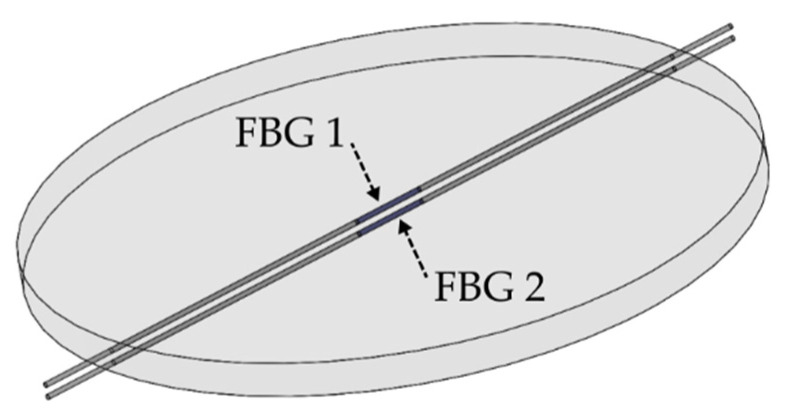
Structure of the silicone rubber diaphragm with two inserted FBGs.

The liquid level monitoring system is made of two FBGs that are inserted into a silicone rubber diaphragm, as shown in Figure 1. To make the diaphragm with FBGs, a mold was created, in which FBGs were positioned in opposite directions, considering the diaphragm bending’s neutral line as a reference; then silicone rubber was applied. To ensure distance, the two FBGs were inserted into holes drilled in the mold wall. After FBGs were positioned, the resin was taken to the greenhouse under 60 °C for 2 h and then cured for 24 h. This resulted in a diaphragm about 2.2 mm thick with 45 mm in diameter. FBGs were approximately 0.8 mm distant from each other.

For sensor analyses, the sm125 FBG interrogator (Micron Optics, Atlanta, GA, USA) with 1 pm resolution was used to acquire the reflected spectra.

**Figure 2:**
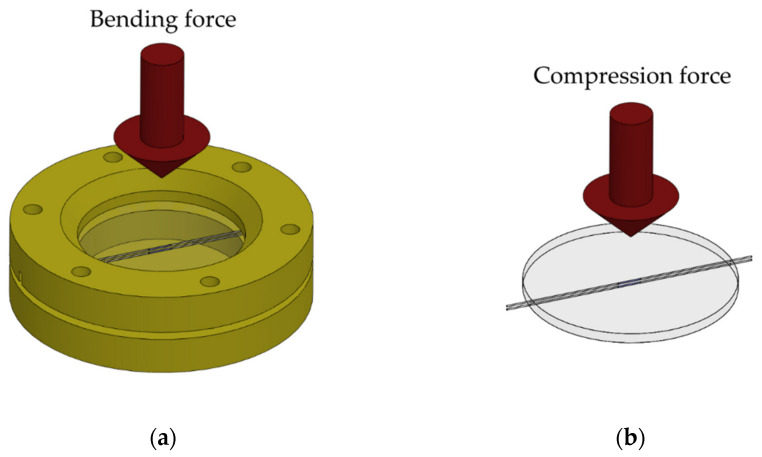
(**a**) Schematic representation of the experimental setup used for bending test characterizations. (**b**) Schematic representation of the experimental setup used for compression test characterizations.

First, the temperature was characterized in the 1/400 ND greenhouse (Ethik Technology, São Paulo, Brazil), ranging from 23.5 °C to 45 °C, to obtain the temperature sensitivity of each FBG. Then, the bending force was characterized by applying calibrated weights to the diaphragm attached to the support (Figure 2a). Conversely, compression force was characterized by supporting the diaphragm alone on a flat surface and applying force with calibrated weights (Figure 2b).

The details of the support built for fixing the diaphragm for the tests are presented in Appendix A.

**Figure 3:**
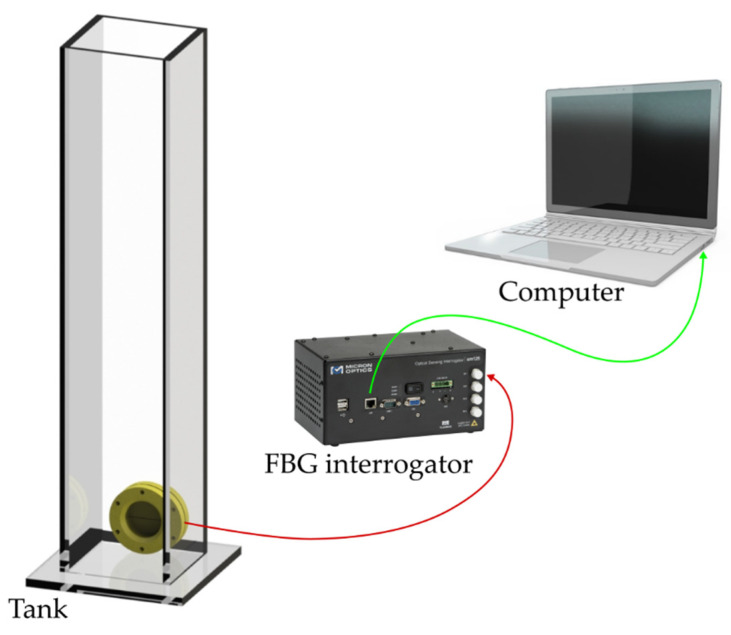
Schematic representation of the experimental setup used for liquid level test characterizations.

As shown in Figure 3 for liquid level characterization, the diaphragm was placed on the support and sealed with silicone to ensure that only one of the diaphragm’s surfaces would be exposed to hydrostatic pressure. Then, the diaphragm was secured on a nylon bar to be put inside the tank. The tank was 1185 mm high, 85 mm wide, and 90 mm deep.

## 3. Results and Discussion

**Figure 4:**
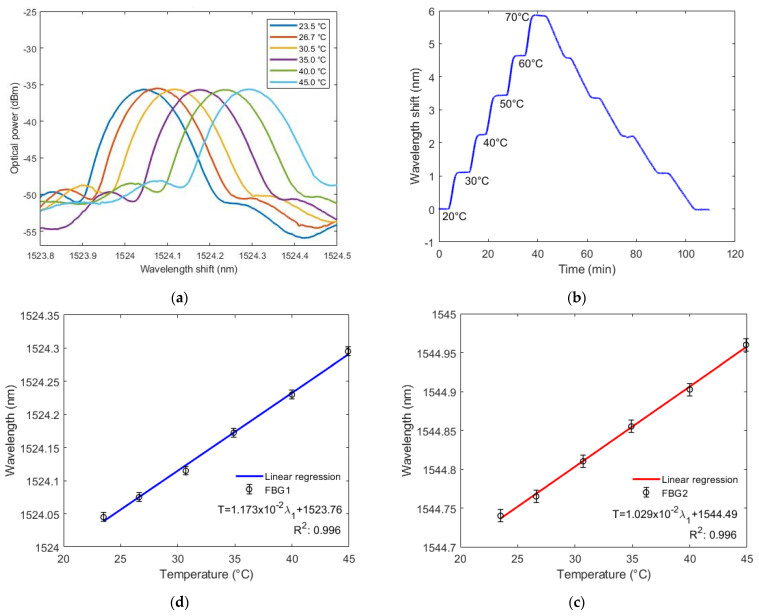
(**a**–**c**) Bragg wavelength shift as a function of the temperature for FBG1; (**d**) Bragg wavelength shift as a function of the temperature for FBG2.

A temperature characterization was conducted in which sensors were put inside a greenhouse with a 23.5 °C to 45 °C temperature variation. After reaching 25 °C, the temperature rose in 5 °C each time until 45 °C. Figure 4a shows the reflected spectra of FBG1 under different temperatures, where it is possible to observe a linear wavelength shift as a function of time. In addition, the temperature response under heating and cooling processes at a larger range is also investigated and shown in Figure 4b for FBG 1, where a temperature variation from 20 °C to 70 °C was applied to the FBG for 110 min. It is also possible to observe a linear variation with negligible hysteresis between heating and cooling processes. 

Figure 4c,d show a variation of Bragg’s wavelength as the temperature increases (from 23 °C to 45 °C) for both FBGs. The standard deviation of the performed tests is approximately 6.0 pm for FBG 1 and 8.0 pm for FBG 2. From the curve slope, the temperature variation sensitivity of FBG1 is 11.728 pm/°C and the sensitivity of FBG2 is 10.286 pm/°C. The determination coefficient (*R*^2^) is defined as the proportion of the variation of one variable (temperature in this case) from the independent variable (wavelength shift in this case). For the temperature tests, the *R*^2^ was 0.99585 for FBG1 response and 0.99620 for FBG2 response. Both FBGs had higher temperature sensitivity than usual for uncoated FBGs (9.75 pm/°C [26]). This is caused by a thermal expansion of the diaphragm material, resulting in more strain (or shift) in the FBGs, besides the shift indicated by Equation (2). However, inserting FBGs in the silicone rubber diaphragm did not increase sensitivity as much as in other materials, such as polyurethane (19.5 pm/°C) and nitrile rubber (49.8 pm/°C). It was also slightly lower than in the same material (PDMS) (12.3 pm/°C), shown in [16].

To experimentally verify the proposed system, we conducted bending, compression, and liquid level tests. In the bending test, calibrated weights were used to control the force that was applied to the diaphragm. Considering gravity as 9.81 m/s^2^, forces between 0 N and 4.9050 N (500 g) were applied. A weight of 0.4905 N (50 g) was added to each of the first two steps. After that, for the following force applications, weights of 0.9810 N (100 g), were added until the last force value. The temperature was set at room temperature and was kept constant. To show sensor repeatability, the experiment was repeated twice with increased force.

**Figure 5:**
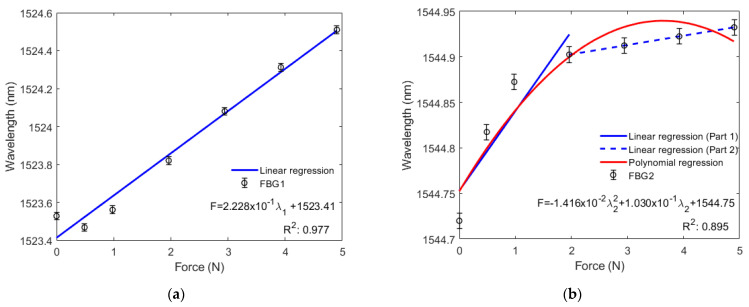
(**a**) Bragg wavelength shift as a function of the bending force for FBG1; (**b**) Bragg wavelength shift as a function of the bending force for FBG2.

Figure 5a shows that the wavelength of FBG1 tends to increase linearly with greater force exerted on the diaphragm, where a standard deviation of 20.5 pm was obtained in an analysis of three consecutive tests. Figure 5b shows that the wavelength of FBG2 tends to increase nonlinearly with 10.8 pm of standard deviation. Thus, from the inclination of the curve in Figure 5a, we found that the sensitivity to force of FBG1 is 222.760 pm/N. However, since FBG2 does not have a linear trend, its sensitivity to force was divided into two parts. Figure 5b shows that sensitivity between 0 N and 1.9620 N (200 g) is 87.520 pm/N and sensitivity between 1.9620 N (200 g) and 4.9050 N (500 g) is 10.194 pm/N. Thus, we obtain a system with a resolution capacity of 4.489 mN for the FBG1, 11.426 mN and 98.097 mN, for the first and second ranges presented previously for the FBG2. The insertion of two FBGs in the diaphragm resulted in a system with greater linearity, because the two FBGs (0.99453) had a higher adjusted determination coefficient (*R_a_*^2^) than the *R*^2^ with FBG1 (0.97684) and FBG2 (0.89461) alone.

Figure 5b also shows a nonlinear response for FBG2. This might have been caused by the difference between the Young module of silica fiber and the silicone rubber diaphragm, since the Young silicone rubber module (0.8 MPa [16]) is smaller than the fiber module (70 GPa [31]). The fiber restricts the diaphragm from transverse elongation by exerting greater force over it and thus reduces stress in the diaphragm and FBG regions. The nonlinear response may also be caused by the material’s anisotropy. When the diaphragm receives greater force, it can suffer a transverse deformation, since the stress around the main plane of the tensor to which it is subjected may be higher [31].

Compression tests were also conducted at constant room temperature, and calibrated weights between 0 N and 49.05 N were used to accurately control the force applied to the diaphragm. For each step, 9.81 N were added.

**Figure 6:**
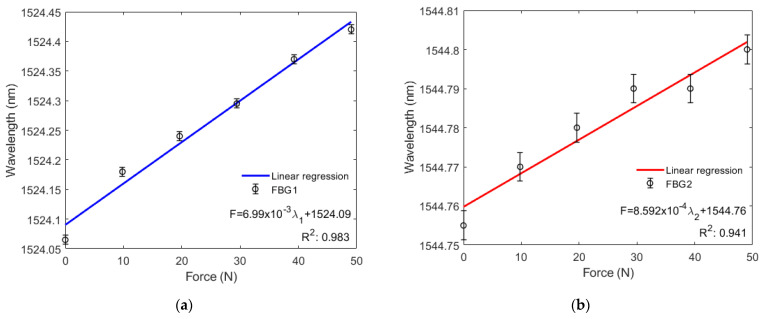
(**a**) Bragg wavelength shift as a function of the compression force for FBG1; (**b**) Bragg wavelength shift as a function of the compression force for FBG2.

Figure 6a,b show that the wavelengths of FBG1 and FBG2 increase linearly with greater force applied to the diaphragm, where standard deviations of 7.6 pm and 3.8 pm were found for FBG1 and FBG2, respectively (in three consecutive tests). This is because the axial compression applied on one of the diaphragm’s surfaces results in a radial distortion of the rubber, which then passes to the FBGs. As a result, the force sensitivity obtained from the curve inclinations in Figure 6a,b are 6.99000 pm/N for FBG1 and 0.85918 pm/N for FBG2. Thus, a system with a resolution capacity of 143.06 mN for FBG1 and 1163.90 mN for FBG2 was obtained. Regarding linearity, the *R*^2^ of the response of both FBGs (0.97142) is lower than the *R*^2^ of FBG1 (0.98271) and higher than that of FBG2 (0.94123).

**Figure 7:**
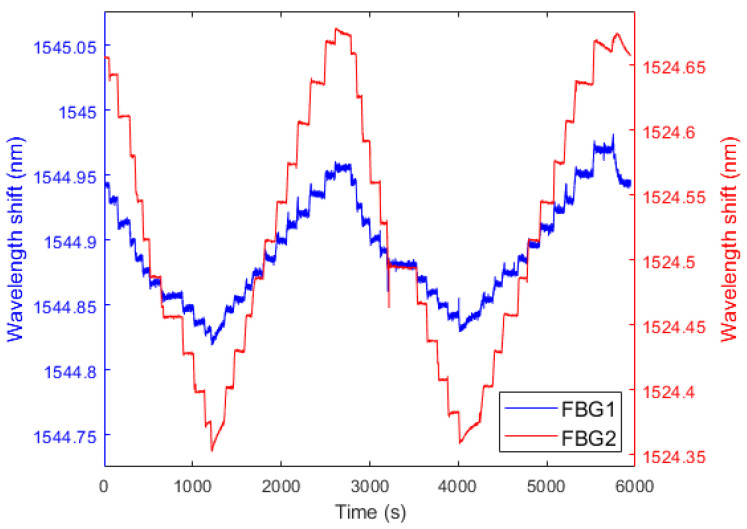
Temporal responses during the rise and fall of the liquid level for FBG1 and FBG2.

The water level test was conducted in two stages at constant room temperature. First, the water level increased from 67 mm to 1100 mm, increasing in steps of 100 mm after reaching the 100 mm level. After the first stage ended, the water level decreased from 1100 mm back to 67 mm. For test reliability, in each test, the level sensor was tested twice with increasing and decreasing levels. Figure 7 show the reflected spectra of FBG1 and FBG2, respectively, in the two stages of increasing and reducing the tank’s liquid level.

**Figure 8:**
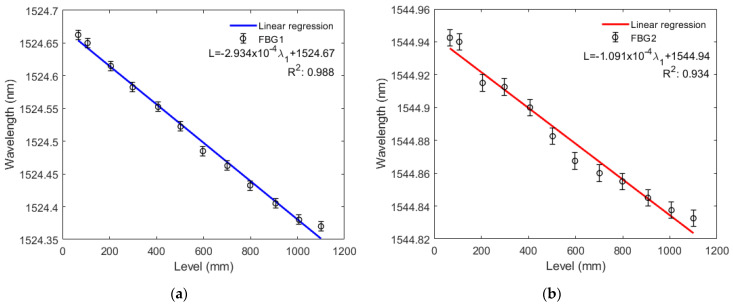
(**a**) Bragg wavelength shift as a function of the tank level rise for FBG1; (**b**) Bragg wavelength shift as a function of the tank level rise for FBG2.

**Figure 9:**
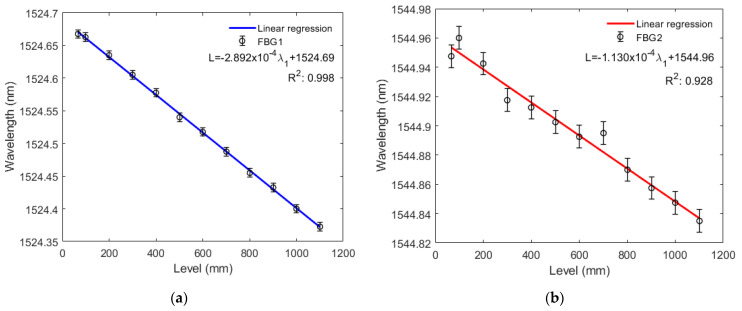
(**a**) Bragg wavelength shift as a function of the tank level reduction for FBG1; (**b**) Bragg wavelength shift as a function of the tank level reduction for FBG2.

Figure 8a,b and Figure 9a,b show that both FBG1 and FBG2 wavelengths tend to decrease linearly with higher liquid levels in the filling and emptying phase. For the liquid level increase, standard deviations of 6.5 pm and 7.7 pm were obtained for FBG1 and FBG2, respectively, whereas standard deviations of 7.2 pm and 5.0 pm were found in the decreasing liquid level, for FBG 1 and FBG 2, respectively. Thus, for the increasing variation of liquid level, sensitivity is −0.29339 pm/mm to the variation of FBG1 level and −0.10907 pm/mm to the variation of FBG2 level. The system showed greater linearity with two FBGs, since the *R*^2^ of the response of both FBGs (0.99163) is higher than that of FBG1 *R*^2^ (0.98848) and FBG2 *R*^2^ (0.93408) alone. Conversely, for the decreasing variation of liquid level, sensitivity is −0.28916 pm/mm to the variation of FBG1 level and is −0.11296 pm/mm to the variation of FBG2 level. With two FBGs in the diaphragm, sensor linearity was similar to that of the compression test, thus resulting in a system with greater linearity than that with FBG2 alone and lower linearity than that with FBG1 alone, since the *R*^2^ of the regression using both FBGs (0.99805) is lower than the FBG1 *R*^2^ (0.99819) and higher than the FBG2 *R*^2^ (0.92844). This can be related to minor deviations in the embedment condition, e.g., the fiber is not perfectly straight and pre-strained. With the results of this and previous tests, we found that FBG2 is not as stable as FBG1, this can be solved by replacing FGB2. Therefore, we can have a system with the capability of estimating liquid level and other parameters mentioned before.

Based on the sensitivities obtained in the level tests, it was possible to obtain a system with a resolution capacity of 3.408 mm for the FBG1 and 9.168 mm for the FBG2 for increasing liquid level variation, and 3.458 mm for the FBG1 and 8.853 mm for the FBG2 for decreasing variation.

## 4. Conclusions

This paper proposed the development and characterization of a liquid level sensor based on a pair of fiber Bragg gratings (FBGs), embedded in a circular silicone rubber diaphragm (PDMS). The sensor was experimentally tested under constant temperature, and we observed its response in various tests. Bending tests used force resulted in a sensitivity of 222.760 pm/N for FBG1. For FBG2, sensitivity was 87.520 pm/N between 0 N and 1.9620 N, and was 10.194 pm/N between 1.9620 N and 4.9050 N. The tests also showed that with two FBGs in the diaphragm, the system had 1.81% higher linearity than that with FBG1 only. It was 11.17% higher than that with FBG2 only, since using two FBGs (0.99453) increased the adjusted determination coefficient (*R_a_*^2^). Thus, we also obtain a system capable of detecting a minimum force of 4.489 mN for bending efforts.

In compression tests, force sensitivities of 6.99 pm/N and 0.86 pm/N were obtained, respectively to FBG1 and FBG2. Regarding linearity, the response of both FBGs in a combined analysis was lower than FBG1 and higher than FBG2. Thus, a system with a minimum force detection capacity of 143.06 mN for compression efforts was obtained.

For the liquid level increase, the sensitivity to level variation was −0.29339 pm/mm for FBG1 and −0.10907 pm/mm for FBG2. The linearity of the combined responses of FBGs 1 and 2 (0.99163) was higher than in FBG1 and FBG2 individually analyzed. Conversely, for the decreasing variation of liquid level, sensitivity was −0.28916 pm/mm to the variation of FBG1 level and −0.11296 pm/mm to the variation of FBG2 level. Combined FBG system linearity was higher than in FBG2 and FBG separately. With this configuration, it was possible to obtain a system with a minimum level detection capability of 3.408 mm.

Temperature characterization results in temperature sensitivity of 11.728 pm/°C for FBG1 and 10.286 pm/°C for FBG2. Both FBGs had higher temperature sensitivity than usual for uncoated FBGs (9.75 pm/°C). However, when compared with polyurethane, FBG1 and FBG2 decreased 39.86% and 47.25% in temperature sensitivity, respectively. When compared with nitrile rubber, FBG1 decreased by 76.45% and FBG2 decreased by 79.35%. Therefore, the proposed system is a feasible method for multiparameter sensing using diaphragm-embedded sensors, where Table 1 shows the comparison of the proposed sensor for temperature, force, and liquid level assessment, with sensors previously proposed in the literature. The comparison is performed as a function of the sensitivity and linearity of each parameter. Although some sensors presented higher sensitivities for the analysis of each parameter, it is important to mention that none of these sensors presented the possibility of measuring all parameters in a single system. In addition, the possibility of measuring temperature with other parameters (such as force and liquid level) leads to the capability of an automatic temperature compensation, without the need for an additional FBG as shown in [31].

Future studies include the application of this sensor in oil tanks and the investigation of the material’s fatigue under several cycles. They serve to analyze the behavior of the sensor when exposed to another type of liquid, and also to estimate the fatigue life of the sensor and analyze the signal degradation of the FBGs.

## Figures and Tables

**Table 1:** Comparison between diaphragm-embedded FBG sensors for multiparameter assessment.

Reference	Temperature		Force/Pressure		Liquid Level	
	Sensitivity	Linearity	Sensitivity	Linearity	Sensitivity	Linearity
This work	11.73 pm/°C	0.996	6.99 pm/N	0.983	−0.29 pm/mm	0.992
[35]	Not reported	Not reported	175.5 pm/kPa	0.999	1.62 pm/mm	0.999
[31]	43.2 pm/°C	0.999	Not reported	Not reported	2.74 pm/mm	0.999
[34]	79.7 pm/°C	0.955	100.7 pm/kPa	0.997	Not reported	Not reported

**Figure A1:**
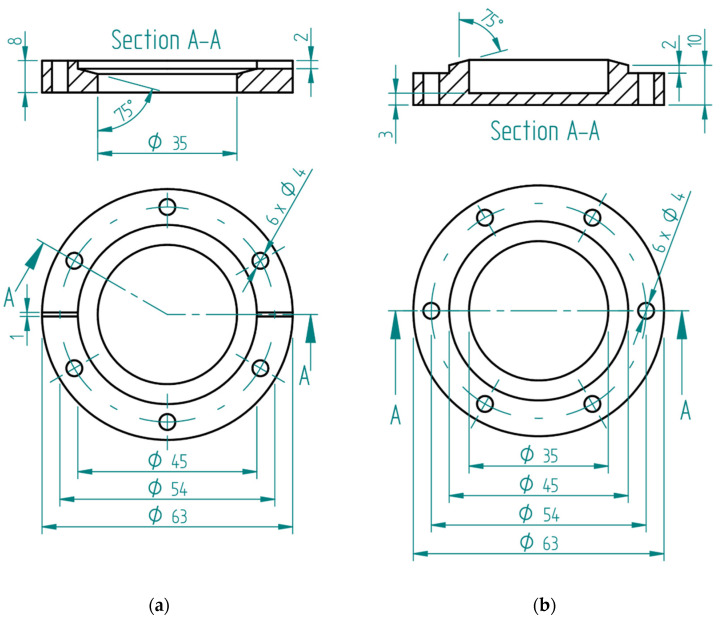
(**a**) Structure of the front of the diaphragm support; (**b**) Structure of the back of the diaphragm support.

## Appendix A

The diaphragm’s support is manufactured with Polylactic acid (PLA) and has two parts. As shown in Figure A1a, the front of the support is 8 mm thick with 63 mm in diameter, with a central hole of 35 mm in diameter, which allows contact between the diaphragm and the liquid. In the upper part, a 2 mm deep and 45 mm diameter undercut was created. At the bottom of the undercut (diaphragm support base), a negative angle of 15° was made for the diaphragm to fit, so that the surface exposed to the liquid would become convex. A 2 mm deep and 1 mm wide groove was also made in the radial direction of the support to hold the optical fiber that reaches the diaphragm.

As shown in Figure A1b, the second part of the support is also 8 mm thick with 63 mm in diameter and has a central undercut of 35 mm in diameter and is 5 mm deep. For the diaphragm to be pressed on the front, a 2 mm high ledge was made, the top was positioned at a positive 15° angle. In both parts of the support, six 4 mm holes were drilled to secure the sensor in place.
